# Experimental System Design and Modelling of EGFR Extracellular Domain and Its Mutant Binding to Antibody Interacting Partner

**DOI:** 10.3390/ijms26083594

**Published:** 2025-04-11

**Authors:** Feyzanur Erdemir, Bertan Koray Balcioglu, Tugba Arzu Ozal Ildeniz, Ozge Can

**Affiliations:** 1 Department of Biomedical Engineering, Institute of Natural Sciences, Acibadem Mehmet Ali Aydinlar University, Istanbul 34752, Türkiye; 2 Medical Biotechnology Unit, Life Sciences, TUBITAK Marmara Research Center, Gebze 41470, Türkiye; 3 Department of Biomedical Engineering, Faculty of Engineering and Natural Sciences, Acibadem Mehmet Ali Aydinlar University, Istanbul 34752, Türkiye; tugba.ildeniz@istinye.edu.tr

**Keywords:** cancer, cetuximab, epidermal growth factor receptor, molecular modeling, R497K mutation

## Abstract

The EGFR pathway is activated by ligand binding, and EGFR overexpression is linked to malignancies like colorectal and head and neck cancer. This pathway is targeted by monoclonal antibodies such as Cetuximab; however, drug resistance can arise, frequently because of EGFR gene alterations like mutation, particularly in domain III, which inhibits Cetuximab binding. EGFR and MEGFR (R497K mutated EGFR) plasmids were transfected into Chinese hamster ovary (CHO) cells, which do not express EGFR. Real-time PCR was performed using probes that were specifically developed for the R497K mutation. Furthermore, Cetuximab binding to EGFR and MEGFR was examined using molecular modeling. According to molecular modeling, the R497K mutation modifies the domain III structure, which lowers the binding affinity of Cetuximab. Curiously, Cetuximab also showed binding to MEGFR’s domain IV. Real-time PCR showed that the probes specifically identified MEGFR in transfected CHO cells. The R497K mutation may result in treatment resistance by decreasing Cetuximab binding or increasing competitive ligand binding. Therefore, for individualized treatment, it is essential to find EGFR mutations in patient tumor samples. The R497K mutation may be successfully detected by the designed oligonucleotide probes, allowing for the early identification of potential resistance and directing the development of suitable treatment strategies.

## 1. Introduction

Cancer is among the leading causes of death worldwide. According to the latest statistics, there were 14.1 million new cases and 8.2 million cancer-related deaths all over the world in 2012 [1]. According to 2020 statistic values, 19.3 million cancer cases have risen, and 10 million cancer deaths have happened [2]. Many cancer types are characterized by abnormal cell proliferation, migration, and invasion. Although many treatment methods and drugs have been developed, the outcomes of the drug treatment would depend on the cancer type, grade, and genetic background of the patient. In many types of cancer, i.e., lung, kidney, breast, head and neck, pancreas, bladder, and colon cancer, epidermal growth factor receptor (EGFR) appears to be expressed more than normal on the cell surface [3]. The over-expression of EGFR causes the cell proliferation that is promoted by the activation of the serine/threonine kinase pathways, etc. [4]. This activation is initiated by the binding of epidermal growth factor (EGF) to the extracellular domain of EGFR, which is also the target region of cancer drugs like Cetuximab. Cetuximab has been developed to treat head and neck and metastatic colorectal cancer, as has Panitumumab [5]. These drugs are designed as anti-EGFR antibodies to inhibit the binding of EGF to EGFR [6]. Some cancer patients have resistance to these drugs due to alterations in EGFR. EGFR alterations include the increase in EGFR gene copy number, the relationship between EGFR and specific ligands, and some mutations. These alterations cause normal cells to change into malignant cells [7]. However, the main reason for drug resistance is known to be the mutation in EGFR [8]. According to the literature, somatic mutations are often detected in exons 18, 19, 20, and 21 of the tyrosine kinase domain of the EGFR gene. The most common mutations are found in exon 19 and exon 21. Exon 19 includes more than 20 deletions; the most common is delE746-A750. The L858R point mutation in exon 21 is the second most common mutation [9]. These mutations are found in the tyrosine kinase domain, and there are other mutations like R497K polymorphism, also termed rs2227983, rs11543848, or R521K, which is seen in the extracellular domain of EGFR [10]. R497K polymorphism is the mutation that occurs in external domain of EGFR, and it causes a decrease in ligand binding, tyrosine kinase activation, and growth stimulus [11].

Molecular modeling is a technique that combines theoretical and computational methods to simulate drug-protein interactions [12]. Molecular dynamics (MD) simulation is a computational method used for studying molecular structural changes over time, as well as the interactions between pairs of molecules. Based in classical Newtonian mechanics, this methodology enables the understanding of atom movement and the system’s dynamic behavior [13]. Atoms are defined as point charges established in relation to mass, and the interactions between atoms are modeled by aspects such as Van der Waals forces, chemical bonds, electrostatic interactions, and hydrophobic interactions. Furthermore, electrostatic interactions are modeled using Coulomb’s law [14]. Energy minimization techniques help to know the stable locations of atoms, and MD simulations enable seeing the behavior of the system over time [15]. The simulations based on Newton’s second law make use of various integration techniques to calculate the evolution of atoms in space and time, thus supporting a better understanding of how ambient environmental conditions and temperature impact dynamic processes [16].

Another modeling technique is molecular docking, which is used for the prediction of the conformation and interactions that a ligand adopts when it binds to a protein receptor. This technique examines the processes of binding between ligands and proteins and displays potential drugs that can be analyzed further [17]. Docking simulations calculate the binding position and orientation of the ligand to the protein, yielding a binding score directly linked to the ligand’s binding energy [18]. Docking analyses help figure out the stability of the protein-ligand complex and important interactions in the binding area.

In this study, structural changes in the extracellular domain of EGFR with the R497K mutation, which may be associated with Cetuximab resistance in cancer patients, were investigated via molecular modeling. The behavior of the R497K mutated EGFR (MEGFR) was mimicked by the molecular dynamics method. The effect of the R497K mutation on the binding of the Cetuximab Fab fragment to the extracellular domain of the EGFR was analyzed by molecular docking.

In addition, primer sets were designed to allow the detection of the R497K mutation. Two primer sets, which were referred to as P1 and P2, were designed to detect EGFR and MEGFR, respectively. The primers were thought to be useful for real-time PCR-based detection of EGFR and MEGFR mRNA expression for detecting mutations before drug use in patients.

## 2. Results

### 2.1. Molecular Dynamics Simulation Results

**Figure 1:**
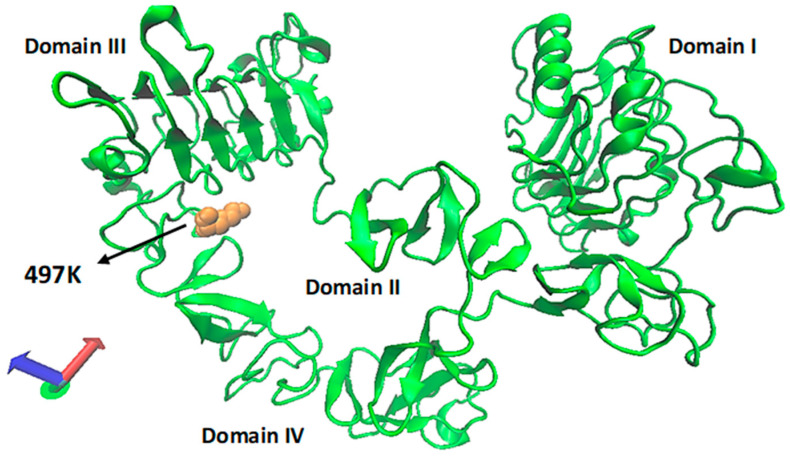
Mutated EGFR external domain structure (green) in tethered form with 497K (orange).

EGFR is composed of four different domain parts. Domain I and domain III refer to L1 or L2 (where L: leucine-rich domain), respectively. Domains II and IV refer to CR1 and CR2 (where CR denotes a high content of cysteine residues), respectively. The R497K mutated EGFR external domain (exMEGFR) is demonstrated in Figure 1. The 497K residue was colored orange (graphical representation: ColorID, VDW).

**Figure 2:**
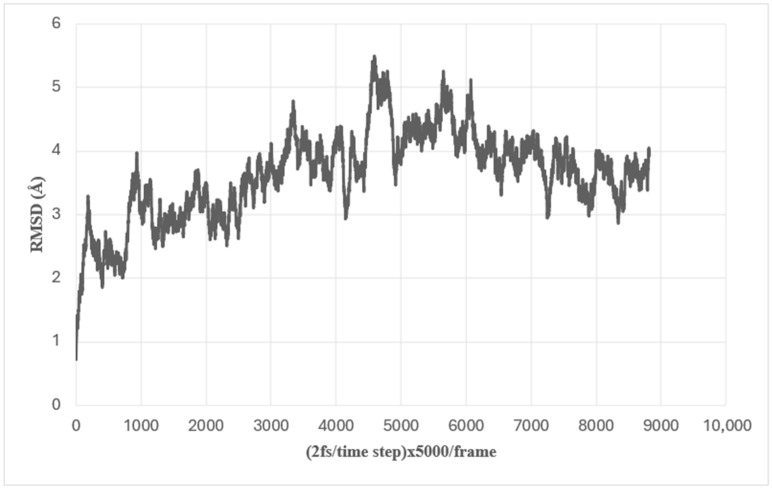
The root mean square deviation (RMSD) of the backbone atoms in the trajectory of exMEGFR during a 90 ns molecular dynamics simulation.

In order to equilibrate the exMEGFR structure, 90 nanoseconds of molecular dynamics simulation was performed. The Root Mean Square Deviation (RMSD) of this simulation was shown in Figure 2, which shows that the RMSD graph shows that the equilibration for exMEGFR was reached.

The molecular dynamic simulation run output trajectories were used for the further docking studies.

### 2.2. Molecular Docking Results

After docking studies, scores of the top 10 best binding sites in Cetuximab-exEGFR (0.5 ns run of experimental PDB structure) and Cetuximab-exMEGFR (90 ns-equilibrated) were given in Table 1 and Table 2. Scores were color-coded from highest to lowest, with red being the highest and yellow the lowest, respectively.

Two different docking studies were performed on the ZDOCK server based on two different approaches. The first docking study was performed based on the defined active site of exEGFR and exMEGFR (Table 1). The active site (ligand binding region) of exEGFR had been known to be the domain III of EGFR from the 1YY9 PDB. Another docking study was performed based on overall exEGFR and exMEGFR structures (Table 2).

From the docking study based on the defined active site of exEGFR and exMEGFR in the 0.5 ns run, it is seen that Cetuximab binds to the domain III of exEGFR and exMEGFR with high scores. It was observed that when the exMEGFR structure was equilibrated in 90 ns, Cetuximab could bind to domain IV as well as to domain III with lower scores.

According to Table 2, for exEGFR in a 0.5 ns run, the drug binds to domain III in the 4th, 6th, and 7th complexes. While exMEGFR were run at 0.5 ns, the binding of the drug to domain III in the 1st and 9th complexes but mostly to domain I and domain II in the other complexes was observed. After a long runtime, i.e., 90 ns, the binding of Cetuximab to domain I/II, and domain IV of exMEGFR mostly was observed with higher scores than in Table 1. It is seen that when docking was performed on the whole external region, Cetuximab can bind to different domains of exEGFR and exMEGFR. As a result, it was observed that in the presence of the mutation, the drug binds to EGFR weakly and the drug can bind to domain IV in addition to the known active site of EGFR (domain III).

**Figure 3:**
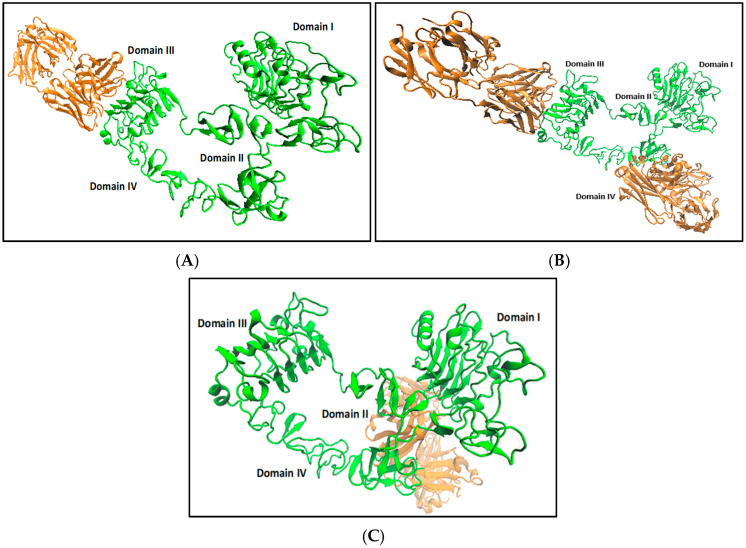
(**A**) Docking of exMEGFR at 0.5 ns (green) and Fab fragment of Cetuximab (orange) (PDB ID: 1YY8); (**B**) docking of exMEGFR at 90 ns (green) and Fab fragment of Cetuximab (orange) (PDB ID: 1YY8) with active site; (**C**) docking of exMEGFR at 90 ns (green) and Fab fragment of Cetuximab (orange) (PDB ID: 1YY8) without active site defined.

The best binding complexes from the docking studies of exMEGFR structures with Cetuximab with/without an active site approach are shown in Figure 3.

When non-equilibrated, exMEGFR (0.5 ns) was used with Cetuximab Fab fragment in a docking study; the drug binds to the domain III of exMEGFR as expected from the exEGFR-Cetuximab complex (Figure 3A). The drug is computed to bind to domain III and domain IV of exMEGFR after a long run (90 ns) with defined active site docking but shows weak binding unlike the binding of Cetuximab to exEGFR (Figure 3B). In Figure 3C, it is seen that the drug can bind to different domains (domain I/II) of equilibrated exMEGFR without a defined active site as well.

### 2.3. Synthesis and PCR Amplification of EGFR and MEGFR

**Figure 4:**
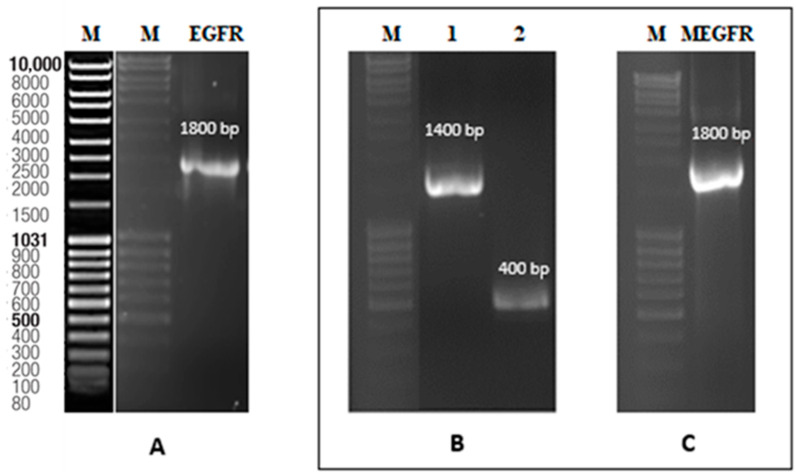
(**A**) 1.2% agarose gel image of the external domain of EGFR. Lane M for marker (MassRuler^TM^ DNA Ladder), lane 2 for EGFR. (**B**) 1.2% agarose gel image of the external domain of MEGFR. Lane 2 for the fragment at the 5′ of the mutation of MEGFR, lane 3 for the fragment at the 3′ of the mutation of MEGFR. (**C**) 1.2% agarose gel image of fusion PCR of MEGFR. Lane 2 for fusion of the fragment at the 5′ of the mutation and the fragment at the 3′ of the mutation.

The synthesized EGFR was successfully amplified by PCR, as shown in Figure 4A. In addition, two segments of the mutated EGFR are presented in Figure 4B. The complete mutated EGFR, constructed through fusion PCR, is illustrated in Figure 4C.

**Figure 5:**
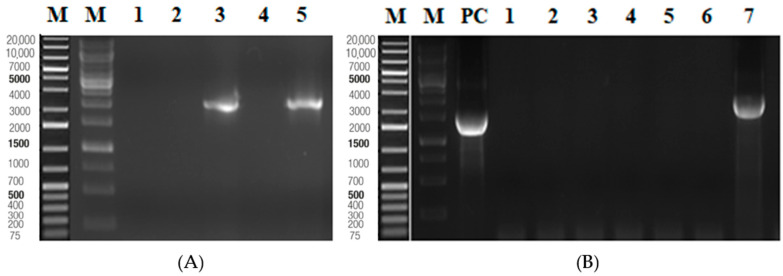
Lane M for marker (Generuler^TM^ 1 kb Plus DNA Ladder). (**A**) 1.2% agarose gel image of EGFR colonies (1800 bp). (**B**) 1.2% agarose gel image of colonies of mutated EGFR (1800 bp).

EGFR and MEGFR encoding genes were amplified by PCR for further cloning experiments. The genes in the pcDNA 3.1 vector were digested with NheI and XhoI restriction enzymes. Their genes were then ligated with the pcDNA3.1 vector and transferred into *E. coli* DH5α cells. The transformant bacterial colonies were analyzed by colony PCR (Figure 5). Two of five colonies contained the EGFR gene (Figure 5A), and only one colony from seven contained the mutated EGFR (Figure 5B).

After colony selection for EGFR and MEGFR, plasmid isolation was performed. The sequence analysis was confirmed for both.

### 2.4. Real-Time PCR Results

**Figure 6:**
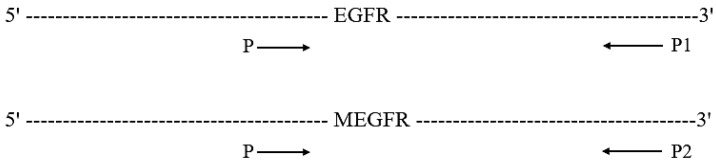
The primers positions, P, P1, and P2 on the EGFR gene.

Real-time PCR was performed with primers specific to EGFR and MEGFR genes (Table 1) to observe the gene expression level in CHO cells transfected with EGFR and MEGFR genes. Forward primer (P) is common for P1 and P2 primer sets. The primer designed specifically for EGFR is referred to as P1. The primer designed specifically for MEGFR is referred to as P2 (Figure 6).

Corresponding forward and reverse primers were used for both EGFR and MEGFR genes to be transfected using CHO cells. Cycle threshold (Ct) and delta Ct values of EGFR P1, P2, and MEGFR P1 and P2 were shown in Table 3. The Ct mean of blank control for primers was approximately ±25 and for GAPDH was approximately ±31. As expected, the Ct value was lower in EGFR-transfected CHO cells using P1. However, the Ct value was higher in EGFR-transfected CHO cells using P2 (Table 3A). Likewise, the Ct value is lower in MEGFR-transfected CHO cells using P2. But the Ct value is higher in MEGFR-transfected CHO cells using P1 (Table 3B).

**Figure 7:**
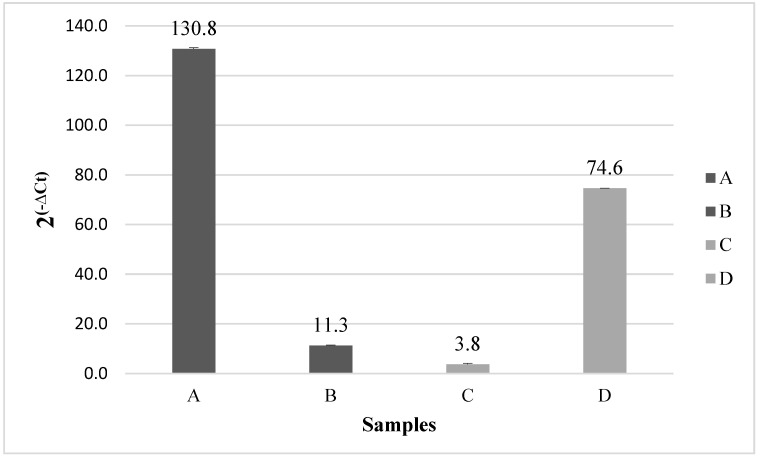
Relative gene expression of EGFR and MEGFR-transfected into the CHO cell line, respectively. (A) EGFR-transfected into CHO cells using the P1 primer set in the qPCR step. (B) MEGFR-transfected into CHO cells using the P1 primer set in the qPCR step. (C) EGFR-transfected into CHO cells using the P2 primer set in the qPCR step. (D) MEGFR-transfected into CHO cells using the P2 primer set in the qPCR step.

After Ct calculation, relative gene expression of EGFR and MEGFR-transfected into CHO cells is shown in Figure 7. Dark columns present the gene expression level of EGFR and MEGFR, respectively, transfected into CHO cells using the EGFR-specific primer (P1) in the qPCR step (Figure 7A,B). Light-colored columns show the gene expression level of EGFR and MEGFR, respectively, transfected into CHO cells using MEGFR-specific primer (P2) in the qPCR step (Figure 7C,D). EGFR gene expression was 130.8 in EGFR-transfected CHO cells using P1, and this amount was 11.3 for MEGFR gene expression. In EGFR-transfected CHO cells using P2, EGFR gene expression was 3.8, and for MEGFR gene expression, it was 74.6. Thus, primers designed specifically for EGFR and MEGFR work to observe the gene expression level of EGFR and MEGFR gene transfected into CHO cell.

## 3. Materials and Methods

### 3.1. Molecular Modeling

The structure file of the EGFR external domain (exEGFR) was extracted from the structure of the exEGFR in complex with the Fab fragment of Cetuximab/Erbitux/IMC-C225 in the Protein Data Bank (PDB) (PDB ID: 1YY9). Mutated EGFR external domain (exMEGFR) was created from the webserver Swiss Model (https://swissmodel.expasy.org, date of access: 1 April 2019) by taking this exEGFR as a template. VMD (v. 1.9) was used to visualize and set up all simulation systems [19]. Energy minimization was performed for both exEGFR and exMEGFR. After the energy minimizations, exEGFR was run at 0.5 ns and exMEGFR was run at 0.5 ns, and an additional 90 ns production run was performed. Then, the docking studies were performed on minimized exEGFR/exMEGFR and equilibrated exMEGFR with the crystal structure of the Fab fragment from the monoclonal antibody, i.e., Cetuximab/Erbitux/IMC-C225 (PDB ID: 1YY8), by using the ZDOCK server (v. 3.0.2).

#### 3.1.1. Molecular Dynamics Simulation

For the molecular dynamics simulations of exEGFR and exMEGFR, structure files were loaded, and then each molecule was put into a separate simulation water box by means of VMD. For these simulations, NAMD Software Version 2.13 was run in parallel with the CHARMM force field parameters. Each system was energy minimized and run at 310 K and 1 atm, with Langevin dynamics and a Berendsen barostat. A 0.5 ns long molecular dynamics run for exEGFR and exMEGFR and a 90 ns long molecular dynamics run for exMEGFR were performed. Trajectory output files of molecular dynamic runs, which provide time dependent displacement output for each atom, were analyzed comparatively. Furthermore, these output files were taken as input files for docking for the binding analysis of Cetuximab to both exEGFR and exMEGFR at various time frames.

#### 3.1.2. Molecular Docking

Docking studies were performed for the binding analysis. For protein-ligand docking, the crystal structure of the Fab fragment from the monoclonal antibody Cetuximab/Erbitux/IMC-C225 (PDB ID: 1YY8) was used as the ligand, and the exEGFR/exMEGFR PDB structure files were used as a receptor. The docking was performed on a protein docking server that is ZDOCK Server (http://zdock.umassmed.edu/, date of access: 15 February 2019). The PDBs of both the receptor and the ligand were loaded into the server, and the server provided a ZDOCK output file, receptor PDB, ligand PDB, top 10 best binding structures, and scores [20]. The obtained complexes were visualized in Visual Molecular Dynamics. The scoring function, which is a mathematical function representing the interaction potential, is important for the basic search algorithm in ZDOCK. After docking, it estimates approximately the binding affinity between the molecules. The scoring function uses a force field to predict the affinities by computing electrostatic interactions and the strength of intermolecular Van der Waals between all atoms of each molecule in the complex. The results were interpreted according to the scores obtained from the scoring, function which gives the highest number for the best bound docking pose ranking in the top 10 predictions [21].

### 3.2. Data Collection and Preparation

The epidermal growth factor receptor gene was synthesized by Sentegen Biotech (Istanbul, Turkiye). The starting gene was in the pTZ57R/t vector. This construct was used as a template to generate the insert containing the restriction enzyme site, Kozak sequence, start codon, and N-terminal His-Tag.

#### 3.2.1. Polymerase Chain Reaction for EGFR and MEGFR

Synthesized EGFR was used as a DNA template. The EGFR gene was amplified with forward primer (F) and reverse primer (R) by using the HotStar HiFidelity Polymerase Kit (Qiagen, Düsseldorf, Germany)shown in Table 4. The PCR procedure includes a 5 min. initial denaturation at 95 °C, 30-cycle amplification step (95 °C for 1 min, 56 °C for 1 min, and 72 °C for 1 min) and 72 °C for a final extension step for 10 minutes. The PCR product was run on 1.2% agarose gel in 0.5 X TAE buffer. The gel was run at 80 volts for 40 min. Then, the bands were monitored with the Azure Biosystem C600 instrument.

**Figure 8:**
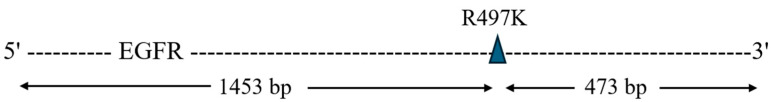
Epidermal growth factor receptor with the R497K mutation site.

To generate the mutated EGFR, a three-step PCR amplification was performed. The HotStar HiFidelity Polymerase Kit was used for this PCR. Part 1, (5′-) of the mutated EGFR, was amplified with EGFR forward primer (F) and R497K reverse primer (MR), and then part 2, (-3′), was amplified with R497K forward primer (MF) and EGFR reverse primer (R) (Table 1). The PCR products were controlled in 1.2% agarose gel. The third amplification step comprises a fusion step followed by an amplification step. The first step of Fusion PCR includes part 1 and part 2 of EGFR, HotStar HiFidelity DNA polymerase, PCR buffer, and nuclease-free water. PCR conditions are 95 °C for initial denaturation and a 7-cycle amplification step (94 °C for 1 min, 63 °C for 4 min). The second step of Fusion PCR includes PCR buffer, nuclease-free water, HotStar HiFidelity DNA polymerase, forward primer (F), and reverse primer (R). The HiFidelity Polymerase Kit protocol was used. The mutation site is shown in Figure 8.

#### 3.2.2. Cloning and Bacterial Transformation

The PCR sample was cut simultaneously with NheI-HF and XhoI restriction enzymes (New England Biolabs (NEB)) according to NEB protocol. All samples were mixed in a 0.5 mL microcentrifuge tube. The tube was placed in the heat block (Techne Dri-Block, DB-2D, UK) at 37 °C for 1 h. The enzymes were inactivated at 80 °C for 20 min. Also, pcDNA 3.1 (+) vector (Invitrogen, Waltham, MA, USA)was digested with the same. For the plasmid digestion, 2 µg of plasmid was used. T4 DNA Ligase (Invitrogen, USA) was used for the ligation process. This protocol was performed for EGFR, pcDNA 3.1 (+) vector, and mutated EGFR, pcDNA 3.1 (+) vector. Insert: Vector Molar Ratio 3:1. The ligation mixture was incubated at 22 °C for 3 h in a water bath, and then the temperature was changed to 65 °C to wait for 10 min. For bacterial transformation, *E. coli* DH5α (Thermo Fisher Scientific, Waltham, MA, USA)was used as a host. The vectors that included the desired gene were transformed into *E. coli* by heat-shock transformation.

**Figure 9:**
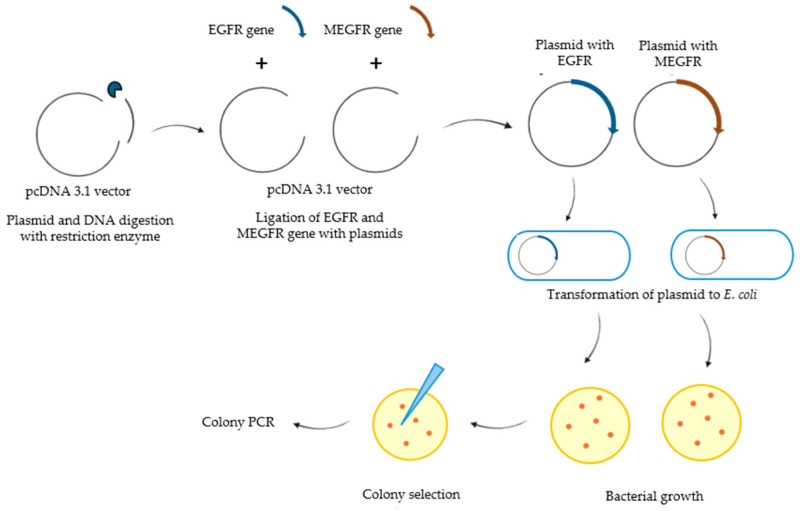
Cloning and bacterial transformation process diagram.

The colony PCR method is the way to control the insertion of the target gene in bacteria after transformation. In the method, 15 µL of distilled water was placed in a 0.5 mL microcentrifuge tube. The colony that grew on the LB/Agar Petri dish (Nunc, Roskilde, Denmark) was picked as a pool. A total of 8 pools of 12 bacteria were performed for EGFR and mutated EGFR. The colonies were mixed with distilled water in a 0.5 mL microcentrifuge tube. The tube was incubated at 95 °C for 3 min, and then it was centrifuged. After that, the supernatant was used as a DNA template. The protocol of the Hotstar Hifidelity Polymerase Kit was applied. After the selection of single colonies, the plasmid was isolated according to the ZymoPURE^TM^ Plasmid Maxiprep (Zymo Research, Irvine, CA, USA) kit protocol. After isolation, the concentration of products was measured in NanoDrop 1000 (Thermo Fisher Scientific, Waltham, MA, USA), and the plasmids were controlled in 1% agarose gel. All processes are shown in Figure 9.

#### 3.2.3. Sequence Analysis

Sequence analysis reactions were carried out using the GenomeLab^TM^ “Dye Terminator Cycle Sequencing” Quick Start Kit (Beckman Coulter, Brea, CA, USA). Reaction results were analyzed with the CEQ 8800 Dye Terminator cycle sequencing automatic sequence analysis system (Beckman Coulter, USA).

#### 3.2.4. Cell Culture and Generation of Transfected Cell Lines

The Chinese hamster ovary cell line (CHO-K1, ATCC^®^ CCL-61™) was used for transfection. For the growth of cells, passage methods were applied according to the data sheet of the cell line. For the transfection method, the Lipofectamine 2000™ Transfection Reagent (Invitrogen, USA) protocol was used. After transfection, the cells were incubated in a CO_2_ incubator (Thermo-Scientific, USA) at 37 °C for 48 h. After 48 h, the supernatant was discarded. Cells were lifted with Trypsin-EDTA (0.25%) (Sigma-Aldrich, St. Louis, MI, USA), and the flask (Corning, Corning, New York, NY, USA)waited at 37 °C in a CO_2_ incubator for 1 min. Then, cells were collected in a 50 mL falcon tube with media that contains 10% FBS. The tube was centrifuged at 1300 rpm for 5 min. After centrifugation, the supernatant was removed, and the pellet was dissolved with DPBS. Then, 1 × 10^6^ cells/mL were put into a 1.5 mL microcentrifuge tube. Finally, the cells were centrifuged at 14,000× *g* for 15 min. The supernatant was discarded, and the pellet was used for RNA isolation.

#### 3.2.5. RNA Isolation and cDNA Synthesis

Direct-zol™ RNA Miniprep Plus Kit (Zymo Research, Irvine, CA, USA)was used to isolate RNA by using the obtained pellet. After RNA isolation, cDNA synthesis was performed with the SensiFast™ cDNA Synthesis Kit (Bioline, London, United Kingdom).

#### 3.2.6. Quantitative Real-Time PCR (qRT-PCR)

For real-time PCR, Syber Green that was provided in iTaq™ Universal SYBR^®^ Green Supermix (Bio-Rad, Hercules, CA, USA)was used. Real-time PCR was obtained in an iCycler iQ™ Real-Time PCR Detection System (Bio-Rad, USA). The experiment was set up with cDNA products of EGFR and mutated EGFR, transfected pcDNA 3.1 (+) vector for blank with two primer sets (Table 4).

## 4. Discussion

The R497K polymorphism located at the extracellular domain of EGFR may be associated with Cetuximab resistance. The effect of the R497K mutation on EGFR has been studied with molecular modeling techniques. According to the results, Cetuximab possibly binds to domains I, II, and IV besides domain III of the exMEGFR. The top ten best binding site complexes for exEGFR and exMEGFR, with and without defined ligand active binding sites of 0.5 ns (not equilibrated) and 90 ns (equilibrated) runs, were given in Table 1 and Table 2. The docking of the Fab fragment of Cetuximab on exEGFR or exMEGFR, performed with the defining ligand binding site, resulted in the binding of Cetuximab to domain III of exEGFR and exMEGFR with a score of approximately 1690. However, the drug also binds to domain IV as well as to domain III of exMEGFR at equilibration (90 ns) with lower scores (approximately 1300).

When the dockings were performed for exEGFR and exMEGFR with the Fab fragment of Cetuximab without defining the ligand binding site, it was observed that the drug can bind to domain III as seen in the 4th, 6th, and 7th complexes with a score of approximately 1700 in 0.5 ns EGFR. In the same conditions, the drug may bind to domain III as well, as seen in the 1st and 9th complexes with a score of approximately 1800, but mostly it binds between domain I and domain II of exMEGFR with the same score. Cetuximab is known to be bound to domain III of EGFR experimentally [22]. This shows that there may exist additional effects other than the computationally predicted interactions apart from the ones just in a simulation-water box, such as additional ions or some other molecules affecting the surrounding of the EGFR/MEGFR in a real situation. We can explore the sampling space better in further modeling trials. Moreover, the binding scores differed in 90 ns (equilibrated) exMEGFR when docking results were analyzed. The binding of Cetuximab to domain IV and domain I/II of exMEGFR mostly was observed with higher scores than in Table 1. Consequently, it was seen that in the presence of the mutation, Cetuximab binds to exEGFR weakly, and the drug can bind to domain IV in addition to the active site of EGFR (domain III). Therefore, if the drug binds to different domains of MEGFR other than domain III, the EGF ligand can bind to domain III to initiate cell proliferation in such cases. Thus, one may develop resistance to the drug. In addition, there are articles in the literature in which other causes of resistance are investigated, as well [23,24]. Even though the binding site of Cetuximab to EGFR is different than the position of the mutation, activation of other possible binding sites other than domain III with the mutation in EGFR may most probably affect the drug binding behavior. As a result, the latter drug resistance may result from other mechanisms than the binding to domain III that is emphasized in the literature. In the study of [23], reduced affinity for the attachment of Cetuximab to domain III in R497K EGFR compared with EGFR was observed. It shows the inability of Cetuximab to inhibit EGFR pathway activation. Also, they claim that the changing from complex-type to unsialylated high-mannose N-glycans because of the amino acid substitution of R497K EGFR resulted in reduced stability and Cetuximab affinity [24].

Based on the modeling results, the presence of mutation in EGFR reduces the binding capacity of the drug, or there is a competition between the drug and EGF for binding to EGFR. As a result, this negatively affects the treatment process in the patient. Therefore, the identification of the mutation in the patient is important for the use of the appropriate drug. Even if there are EGFR mutation detection kits available on the market, to the best of our knowledge, no kit has been developed for the detection of the R497K mutation. Thus, we aimed to design a probe (oligonucleotide) for the diagnosis of this mutation.

The probes are specific for the EGFR and MEGFR genes and the primer sets designed for the detection of either EGFR or MEGFR have been confirmed with real-time PCR experiments. Normally, the most common method for identifying mutations is direct sequencing. Its sensitivity has been proven, but it has never become popular [25]. The reason is that the detection limit reaches approximately 25–30% for mutant DNA [26]. A low level of DNA cannot be seen in direct sequencing. Chong et al. determined EGFR mutations on exons 18, 19, 20, and 21, with the real-time PCR and direct sequencing method. They have tested EGFR mutations with these two methods, and they have shown a higher percentage of tumors with real-time PCR (170 [42.9%] of 396 tumors) than by direct sequencing (151 [36.3%] of 416 tumors) [27]. Some molecular diagnostic kits were already developed for EGFR mutation detection. The Rascreen EGFR RGQ PCR Kit was used for the detection of exon 19 deletions, L858R, L861Q, G719X, S768I mutations, exon 20 insertions, and the Erlotinib resistance mutation T790M in the EGFR gene by using real-time PCR. Angulo et al. detected EGFR mutations on exons 18, 19, 20, and 21 by direct sequencing. They aimed to develop a detection kit for 19 deletions in exon 19, 3 insertions in exon 20, and point mutations (G719X, S768I, T790M, L858R, and L861Q). According to the results, the sensitivity of the real-time PCR based on the EGFR mutation test kit was 95%, which was much higher compared to direct sequencing. The reason for the 5% error margin was because of a false negative due to an insertion in exon 20. This insertion was detected by direct sequencing instead of a test kit. The kit target was restricted to the detection of mutations with a sensitivity of 100% [28]. Even with the next generation sequencing technologies, low quantities of mutated DNA in a tumor sample may yield false negative results, and also the method is relatively expensive. In contrast, real-time PCR shows higher sensitivity, accuracy, and applicability compared to other sequencing methods [29]. In the experiment, the CHO cell line was chosen for transfection as CHO did not express the EGFR gene. Thus, EGFR expression can be confirmed after transfection. The designed probes were used in real-time PCR after expression of EGFR and MEGFR in CHO cells. According to the results, EGFR gene expression is 130.8 (2 ^(−∆Ct)^) when EGFR-specific primer is used in qPCR, while the expression value is 3.8 (2 ^(−∆Ct)^) when MEGFR-specific primer is used. Therefore, these results demonstrated the specificity of P1 for wild-type EGFR. For MEGFR expression, the gene expression value is 74.6 (2 ^(−∆Ct)^) when using the mutated specific primer set (P2) in qPCR, while the gene expression value is only 11.3 (2 ^(−∆Ct)^) when using the EGFR-specific primer (P1). These results demonstrated the presence of mRNA expression of EGFR and MEGFR in transfected CHO cells, known as cells that do not express EGFR.

The real-time PCR primer sets developed in this current work provide a promising new approach to discerning the R497K mutation that has been associated with Cetuximab resistance. Real-time PCR is a highly established, and moreover, a highly sensitive and specific means for detecting mutations with the advantage of clinical use [30]. When compared with more labor-intensive sequencing approaches, real-time PCR offers quick results and would aid in therapeutic decision-making [31].

The unique characteristic of our real-time PCR method is its ability to clearly recognize the R497K mutation in tumor samples with high specificity. The oligonucleotide probes developed in this study were able to clearly differentiate between wild-type EGFR and MEGFR (R497K-mutant EGFR) in transfected CHO cells, providing further evidence that this assay could be useful for clinical laboratory services to detect this mutation early and identify patients for whom Cetuximab therapy is unlikely to be beneficial.

A few limitations should also be considered. First, use of the primer sets was initially assessed on transfected CHO cells under controlled in vitro conditions. It only gives an idea of efficiency and specificity, and until and unless validated with clinical tumor samples, one should not completely rely on it to demonstrate its performance in a clinical setting [32]. Real-time PCR also might not find some other unique EGFR mutations responsible for resistance because it only detects known mutations. For comprehensive mutation profiling, it is recommended to use additional techniques like next-generation sequencing (NGS) [33]. Despite these limitations, our real-time PCR approach offers a quick and cost-effective way of spotting the R497K mutation even ahead of Cetuximab treatment.

In conclusion, the designed system can be applied to clinical samples after performing a carefully designed clinical study. Oligonucleotides designed to detect EGFR mutation status can be applied to patient tumor samples to estimate a possible drug resistance scenario. Both P1 and P2 primer sets can be used to check differences in gene expression levels to decide about the mutation status. For further studies, the modeling of mutated EGFR can be improved by adding membrane and/or including glycosylation sites of EGFR/MEGFR. For the experimental study, mutated EGFR can be expressed, purified, and characterized in terms of glycan profiles to make sure we obtain a similar protein as in native form.

## 5. Conclusions

In this study, binding behavior in MEGFR is observed to be different than in wild-type EGFR. It is computationally seen that there exist other possible binding sites like domain IV in EGFR that Cetuximab can bind to. This simultaneous serving of various domains in MEGFR as binding sites for Cetuximab, makes the binding of EGF possible to domain III, which competes with Cetuximab for binding in the wild type EGFR case.

The real-time PCR primer sets developed in this study have the potential to be used as an easy method for the detection of EGFR mutation before the administration of drugs such as Cetuximab in cancer patients. Further studies on different cancer cell lines and patient tumor samples will be needed to decide the drug resistance status in the presence of the R497K mutation.

## Figures and Tables

**Table 1:** Top 10 best binding sites and scores for Cetuximab-exEGFR and Cetuximab-exMEGFR based on the active binding site option in ZDOCK.

Receptor	Time (ns)		1	2	3	4	5	6	7	8	9	10
EGFR	0.5	Score	1694	1573	1525	1509	1386	1374	1327	1318	1282	1236
		Binding Site	d3	d3	d3	d3	d3	d3	d3	d3	d3	d3
MEGFR	0.5	Score	1623	1542	1499	1367	1321	1221				
		Binding Site	d3	d3	d3	d3	d3	d3				
MEGFR	90	Score	1303	1246	1214	1152						
		Binding Site	d3–d4	d3	d3	d3						

**Table 2:** Top 10 best binding sites and scores for Cetuximab-exEGFR and Cetuximab-exMEGFR based on overall exEGFR and exMEGFR structures.

Receptor	Time (ns)		1	2	3	4	5	6	7	8	9	10
EGFR	0.5	Score	1809	1795	1777	1725	1706	1694	1685	1681	1672	1662
		Binding Site	d4	d1–d2	d1–d2	d3	d1–d2	d3	d3	d4	d2–d4	d1–d4
MEGFR	0.5	Score	1966	1917	1879	1862	1843	1824	1786	1778	1772	1764
		Binding Site	d3	d1–d2	d1–d2	d1–d2	d1	d1–d2	d1	d1	d3	d4
MEGFR	90	Score	1879	1821	1779	1716	1684	1664	1651	1641	1637	1635
		Binding Site	d1–d2	d1–d2	d1–d2	d1–d2	d4	d3	d1	d4	d4	d4

**Table 3:** EGFR/MEGFR mRNA expression level monitoring. (**A**) EGFR expression. (**B**) MEGFR expression.

**(A)**						
	**Identifier**	**Av. Ct**	**Identifier**	**Av. Ct**	**ΔCt**	**2^−ΔCt^**
CHO cell	GAPDH	24.9	P1	31.5	6.6	0
CHO cell transfected with pcDNA 3.1 vector	GAPDH	25.8	P1	28.5	2.7	0.2
CHO cell transfected with EGFR/pcDNA 3.1 vector	GAPDH	23.5	P1	16.5	−7	130.8
CHO cell	GAPDH	24.9	P2	32.5	7.6	0
CHO cell transfected with pcDNA 3.1 vector	GAPDH	25.8	P2	31.1	5.3	0
CHO cell transfected with EGFR/pcDNA 3.1 vector	GAPDH	23.5	P2	21.6	−1.9	3.8
(**B**)						
	**Identifier**	**Av. Ct**	**Identifier**	**Av. Ct**	**ΔCt**	**2^−ΔCt^**
CHO cell	GAPDH	24.9	P1	31.5	6.6	0
CHO cell transfected with pcDNA 3.1 vector	GAPDH	25.8	P1	28.5	2.7	0.2
CHO cell transfected with MEGFR/pcDNA 3.1 vector	GAPDH	22.6	P1	19.1	−3.5	11.3
CHO cell	GAPDH	24.9	P2	32.5	7.6	0
CHO cell transfected with pcDNA 3.1 vector	GAPDH	25.8	P2	31.1	5.3	0
CHO cell transfected with MEGFR/pcDNA 3.1 vector	GAPDH	22.6	P2	16.4	−6.2	74.6

**Table 4:** Designed primers for PCR and qPCR.

Primer Name	Sequence (5 > 3)	Base Pair (bp)	Temperature (°C)
EGFR Forward primer (F)	TTATGCTAGCGCCGCCACCATGCACCATCATCATCACCATCACCACCTGGAAAAGAAAGTTTGCC	68	76
EGFR Reverse primer (R)	ATGGGCCTAAGATCCCGTCCTGACTAGCTCGAGTCAA	37	73.9
R497K EGFR Forward primer (MF)	CCAAAATTATAAGCAACAAGGGTGAAAACAGC	32	59
R497K EGFR Reverse primer (MR)	GCTGTTTTCACCCTTGTTGCTTATAATTTTGG	32	59
qPCR-Forward primer	CAGCCTGAACATAACATCCTTGG	23	60.6
qPCR-Reverse primer without mutation for P1	CTTGCAGCTGTTTTCACCTCTG	22	60.3
qPCR-Reverse primer with mutation for P2	CTTGCAGCTGTTTTCACCCTTG	22	60.3

## Data Availability

The original contributions presented in this study are included in the article. Further inquiries can be directed to the corresponding author(s).
